# Supplementary material for: Effect of Alkylresorcinols Isolated from Wheat Bran on the Oxidative Stability of Minced-Meat Models as Related to Storage
Source: Antioxidants (Basel). 2024 Jul 30;13(8):930. doi: 10.3390/antiox13080930 (PMC11351659; doi:10.3390/antiox13080930)

Supplementary Material

Effect of Alkylresorcinols Isolated from Wheat Bran on the Oxidative Stability of Minced-Meat Models as Related to Storage

Carolina Cantele [1], Giulia Potenziani [1], Ambra Bonciolini [1], Marta Bertolino [1,†] and Vladimiro Cardenia [1,2,*,†]

[1] Department of Agricultural, Forest and Food Sciences (DISAFA), University of Turin, 10095 Grugliasco, TO, Italy; carolina.cantele@unito.it (C.C.); giulia.potenziani@unito.it (G.P.); ambra.bonciolini@unito.it (A.B.); marta.bertolino@unito.it (M.B.)
[2] AgriForFood Chromatography and Mass Spectrometry Open Access Laboratory, University of Turin, 10095 Grugliasco, TO, Italy
* Correspondence: vladimiro.cardenia@unito.it
† These authors contributed equally to this work.

Figure S1. Molecular structure of alkylresorcinols.

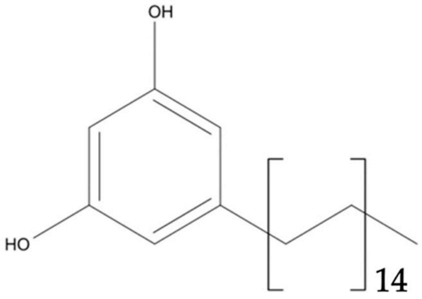

AR15:0

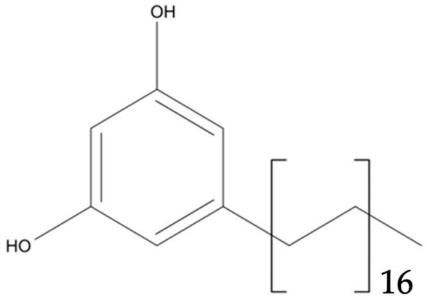

AR17:0

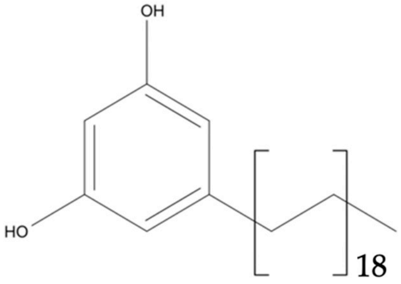

AR19:0

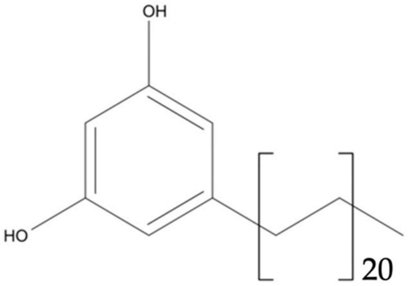

AR21:0

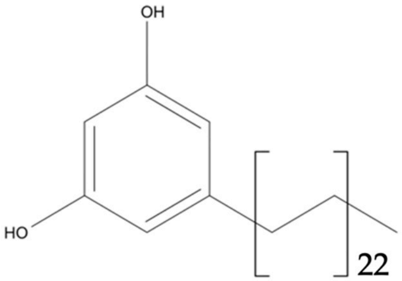

AR23:0

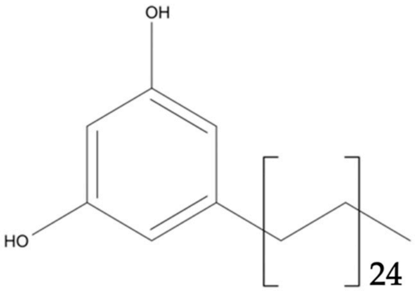

AR25:0

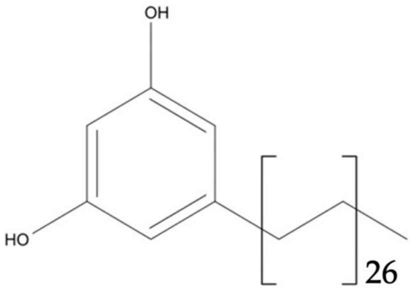

AR27:0

Figure S2. GC/MS spectra of the alkylresorcinols (TMS derivatives) extracted from wheat bran.

AR15:0 TMS derivative

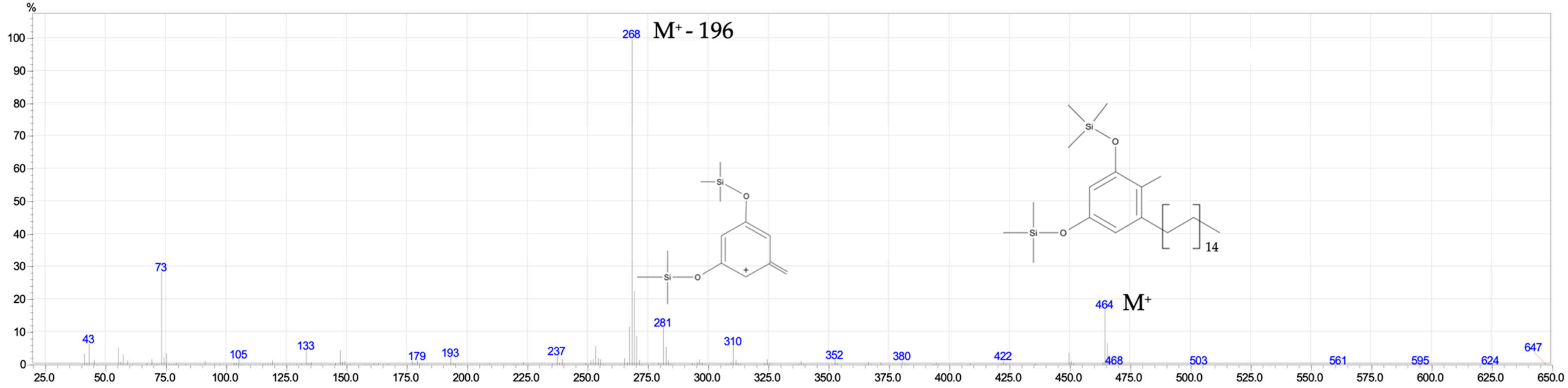

AR17:0 TMS derivative

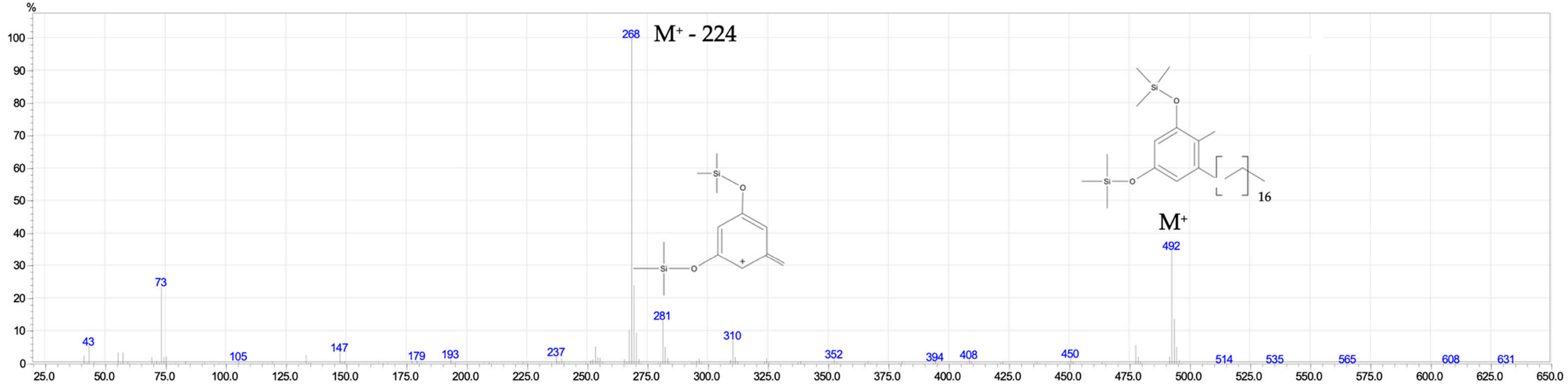

AR19:0 TMS derivative

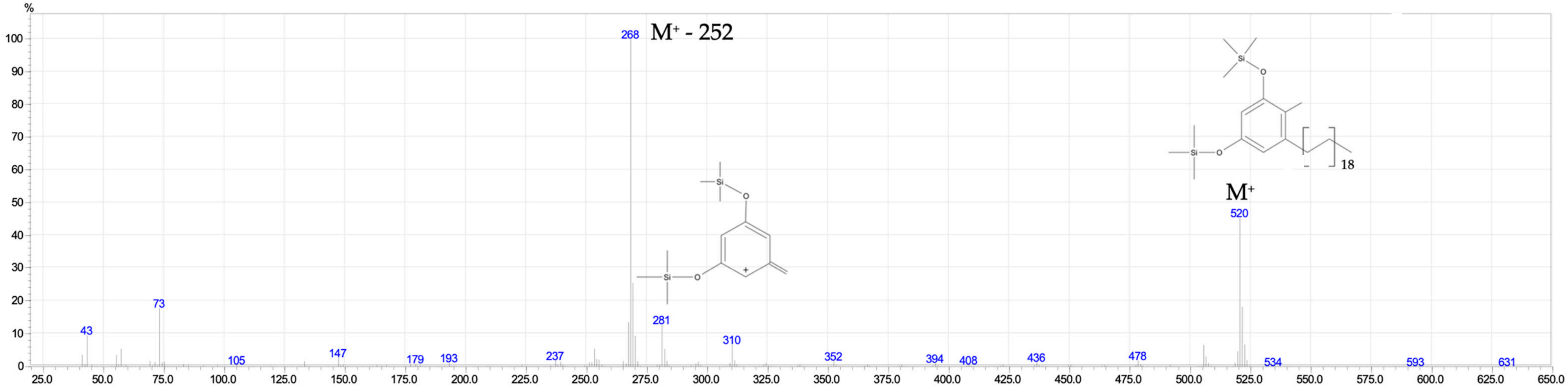

AR21:0 TMS derivative

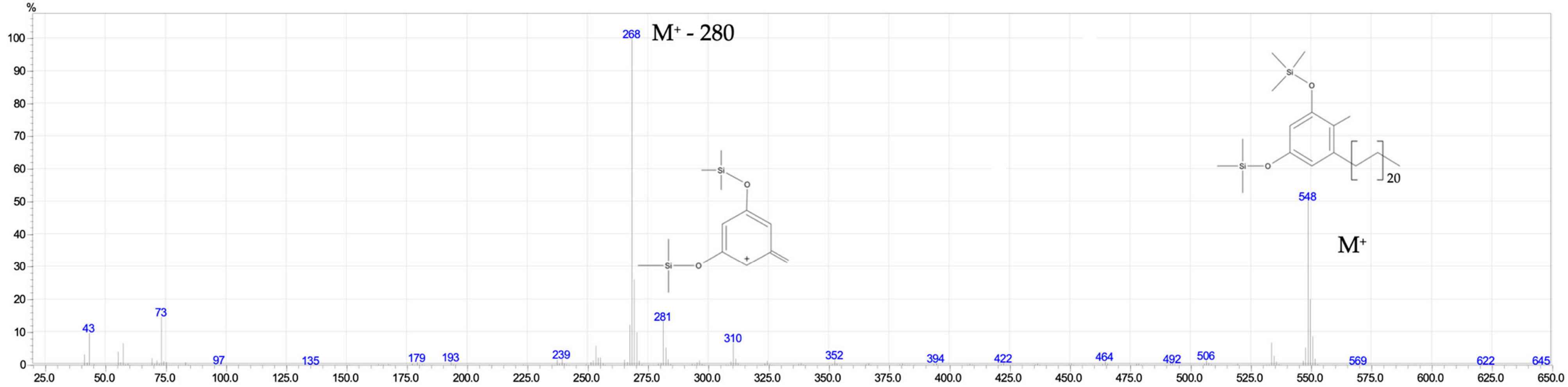

AR23:0 TMS derivative

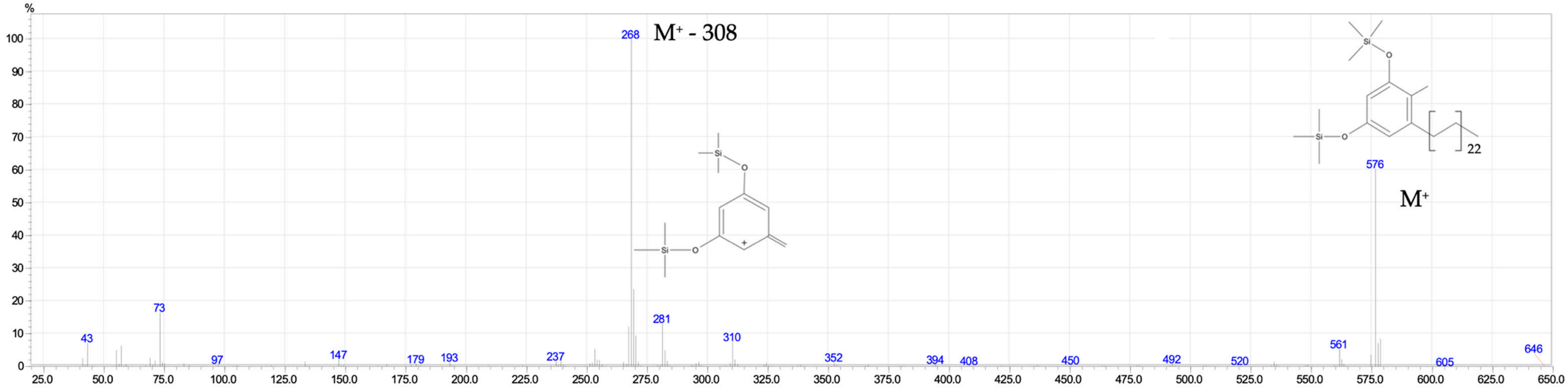

AR25:0 TMS derivative

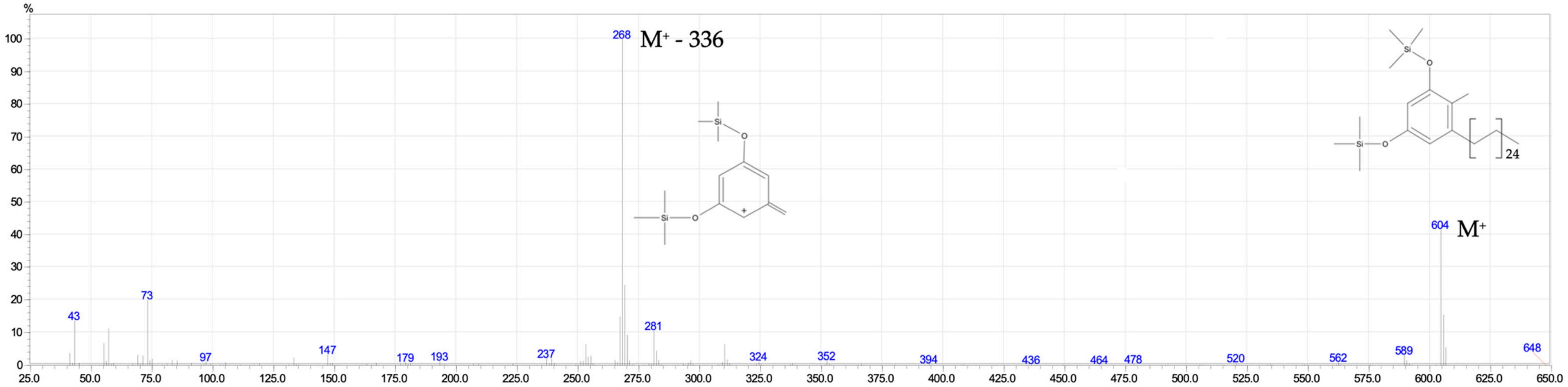

AR27:0 TMS derivative

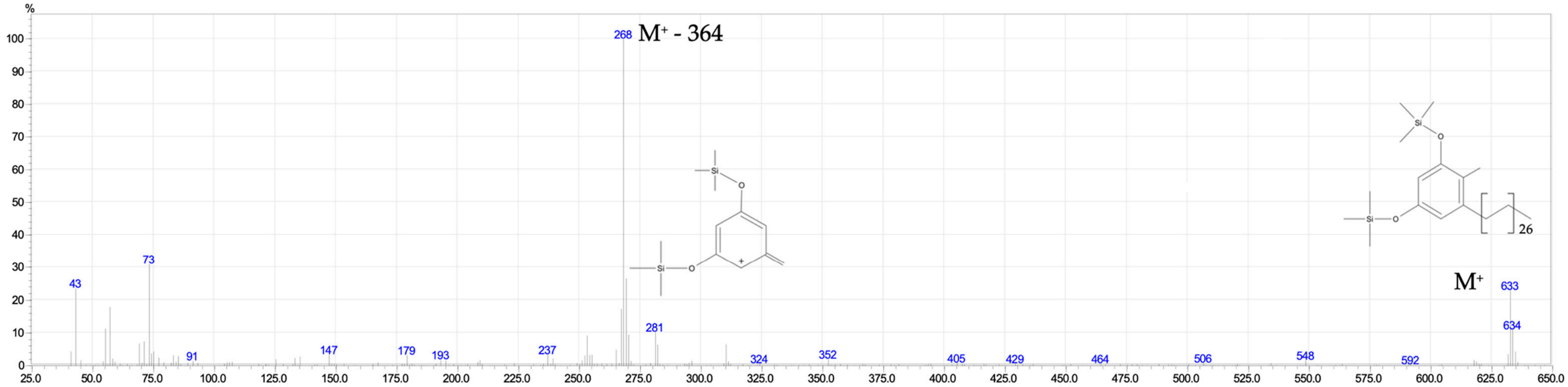

Supplement: Supplementary file 1 [file antioxidants-13-00930-s001.zip › antioxidants-3118775-supplementary.pdf]
